# A Versatile Tool to Predict and Guide RESOLFT Images Based on Photoswitching, Labelling and Optical Properties

**DOI:** 10.1002/cphc.202500780

**Published:** 2026-04-21

**Authors:** Andreas Bodén, Staffan Al‐Kadhimi, Eleonora Uriati, Ilaria Testa, Francesca Pennacchietti

**Affiliations:** ^1^ Department of Applied Physics and Science for Life Laboratory KTH Royal Institute of Technology Stockholm Sweden

**Keywords:** fluorescent proteins, imaging simulation, photoswitching, RESOLFT, super‐resolution microscopy

## Abstract

Reversibly switchable fluorescent proteins (RSFPs) transition many times between dark and fluorescent states under minimal light doses. The photoswitching can happen at different speed, contrast and length, and it is often challenging for users to select the optimal imaging scheme to generate images with high contrast and spatial resolution. Here, we experimentally investigate the photophysical properties of different RSFPs under imaging conditions, together with an in silico exploration of their role in nanoscale image formation. We developed open‐source software that uses measured parameters such as brightness, switching speed, photoswitching fatigue, labelling densities, noise and illumination type to generate the related RESOLFT (reversible saturable/switchable optical fluorescence transition) super‐resolution image. This tool can be used to select optimal imaging schemes for known RSFPs and to guide the rational development of new proteins.

## Graphical abstract

A comparative photophysical study of reversibly RSFPs is coupled to an open‐source simulator to predict RESOLFT image quality from measured brightness, switching rates, contrast, labelling density and illumination scheme. Different image quality metrics enable rational investigation of the imaging parameters, supporting microscope design and probe engineering.

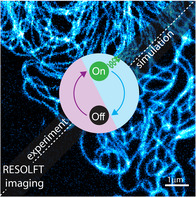

## 1 Introduction

Any imaging method requires fine tuning of a multitude of parameters to achieve optimal image quality. In the field of super‐resolution microscopy, these parameters extend to the photophysical properties of the fluorophores such as brightness, blinking, photobleaching or sample properties such as labelling densities but also imaging system properties such as illumination types, sampling, detector sensitivity and noise [1, 2, 3, 4, 5]. These parameters influence the image formation and have a major impact on the final image quality. Microscopes based on the RESOLFT (reversible saturable/switchable optical fluorescence transition) [6] concept have been demonstrated with reversibly switchable fluorescent proteins (RSFPs) [1, 2, 7] or organic fluorophores [8, 9]. RSFPs can be repeatedly switched between a fluorescent (on) and a non‐fluorescent (off) state, thus adding also switching kinetics and on/off contrast to the collection of system parameters defining imaging performance. The transitions between the states are induced by illumination with specific wavelengths of light. In point scanning [10] and parallelised RESOLFT [11, 12] implementations, spatially patterned illumination coupled with the on/off switching ability of RSFPs create confined nano‐volumes of fluorophores populating the on‐state. The exact size and shape of the resulting emitting volumes depends on the properties of the fluorophores and the illumination scheme which thus determines the quality of the final image. Given the many parameters involved, it is not trivial to assess the imaging strategy that will provide the optimal image quality. A rational pipeline to guide the choice of imaging and RSFP parameters is still missing. Here, we tackle this challenge by providing a comprehensive experimental characterisation of current RSFPs under imaging conditions together with a computational platform to predict and compare RESOLFT image quality from spectroscopic, sample and imaging parameters. Based on our previous theoretical work on predicting Fourier ring correlation (FRC) curves for image quality of RESOLFT data [13], we developed an open‐source software to explore the effect of different fluorophore and optical parameters on the frequency‐domain correlation‐based resolution metric. By feeding the algorithm with the RSFP unique photophysical parameters, such as brightness, switching speed and on/off contrast, we can simulate RESOLFT images and directly evaluate the predicted image quality. By decoupling the influence of individual photophysical parameters, the in silico environment complements the experimental characterisation, where only a limited part of the parameter space can be explored. Overall, the presented photophysical characterisation and RESOLFT image generator software help select optimal imaging settings, aid the development of new RESOLFT microscopes and support probe engineering.

## 2 Results and Discussion

### 2.1 Experimental Characterisation of RSFP for RESOLFT Imaging

**FIGURE 1:**
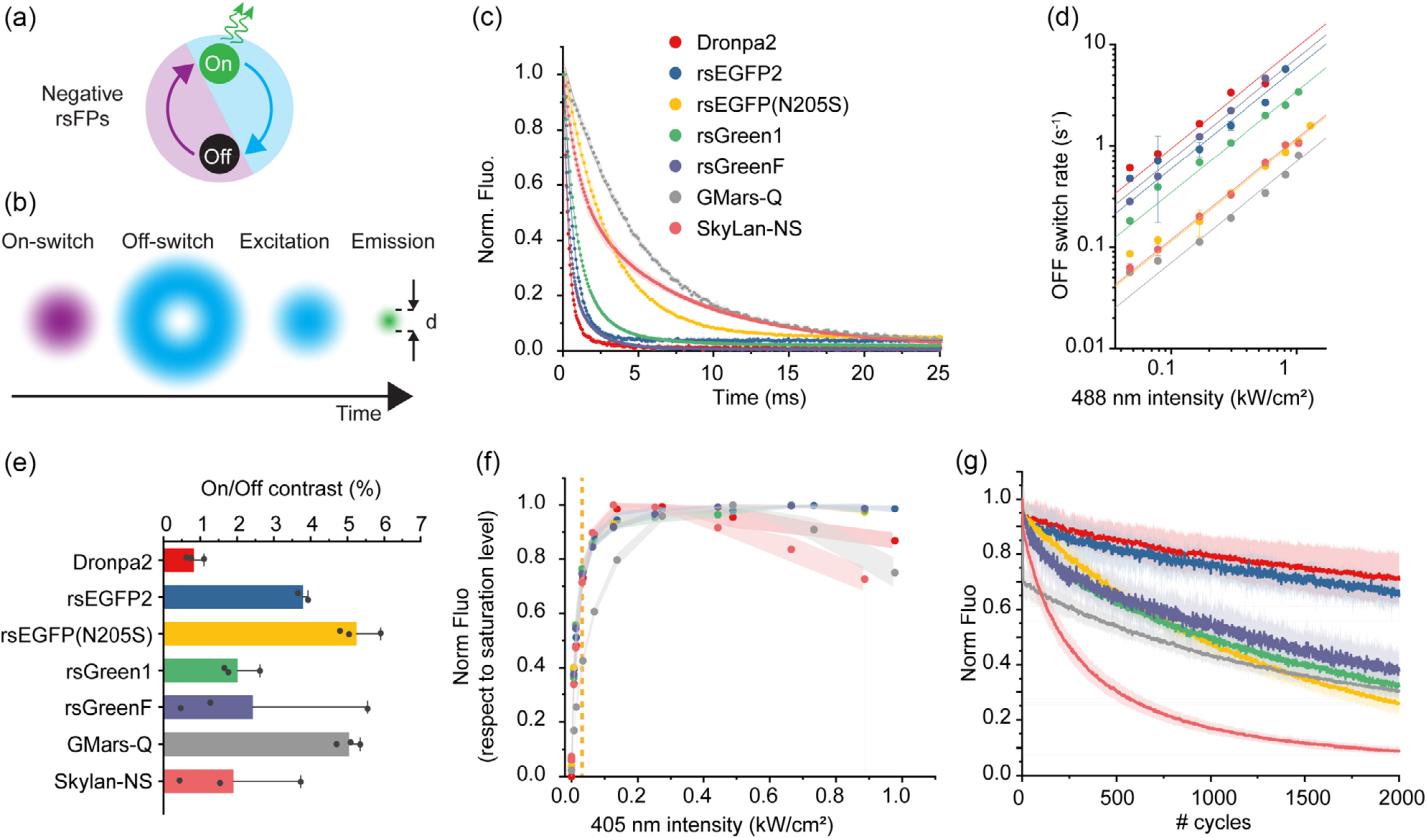
Photophysical characterisation of green negative RSFPs for nanoscale imaging. (a) RESOLFT imaging scheme for a negative switcher and (b) corresponding imaging sequence for it. (c) Representative off‐switching decay for different RSFPs under 0.25 kW/cm^2^ 488 nm illumination (after 0.5 ms of on‐switching with 405 nm light at 0.092 kW/cm^2^). The decay has been collected in cells transfected with either LifeAct (Dronpa2, rsEGFP, GMars‐Q, SkyLan‐NS) or vimentin (rsEGFP2, rsGreen1, rsGreenF). To account for the global decay of the fluorescence upon 488 nm light, the rate is calculated considering the 1/e decay time. For each curve, the mean ± std obtained by averaging 25 switching cycles is reported. (d) Comparison of the off‐switching rate for the different RSFPs, in a range of 488 nm power from 0.025 to 1 kW/cm^2^. The graph also shows the linear fit of the log–log dependency. (e) Residual fluorescence under 0.25–0.3 kW/cm^2^ intensity of 488 nm illumination. The values reported are from three independent experiments (mean ± std). (f) On‐switching dependency to the 405 nm light normalised to the maximum level of on‐switching reached. For each 405 nm light value, the integrated fluorescence under 113 W/cm^2^ of 488 nm light is considered. The time of integration corresponds to the one needed to switch off the fluorescence to 20% of the photoswitchable fraction. Each curve is shown as mean ± std of three repetitions. The dotted yellow line corresponds to the value generally used for imaging. (g) Fatigue curves, meaning the number of cycles sustained by each protein given the same on‐ and off‐switching powers (405 nm at 66 W/cm^2^ and 488 nm at 262 W/cm^2^). For each protein, a cycle is considered the integrated fluorescence over the time needed to have an 80% decrease of the photoswitchable fraction. The measurements are conducted in cell, and each curve is the average (±std) of three to five repetitions in different cells.

Among the different types of RSFPs, we focused our characterisation on green negatively switching RSFPs, in which the excitation wavelength also drives the protein to the off‐state [14] (Figure 1a). The molecular basis of their switching is a *cis*–*trans* isomerisation between the anionic fluorescent *cis*‐form of the GFP‐like chromophore and the dark protonated *trans*‐form [15, 16]. As the most numerous class of RSFPs, it offers a broad sampling of photophysical parameter combinations relevant to nanoscale imaging.

The minimal photophysical parameters that define an RSFP for imaging include photoswitching rates, brightness and fatigue, i.e., the number of switching cycles before irreversible photo‐bleaching (Figure 1b). The off‐switching kinetics describes the rate at which the RSFP population transits from the on‐ to the off‐state under off‐switching illumination, and it is the time limiting step of most RESOLFT image acquisition schemes (0.3–100 ms depending on the illumination intensity). The off‐switching rate depends on the blue light illumination intensity. We can identify Dronpa2 [17, 18], rsEGFP2 [2, 19] and the rsGreen [20] family as the fastest RSFPs of the analysed group with cross sections for the on‐to‐off process higher than 2.5 × 10^−18^ cm^2^ and SkyLan‐NS [21], GMars‐Q [22] and rsEGFP(N205S) [11] as the slowest with cross section for the on‐to‐off process lower than 1 × 10^−18^ cm^2^. If we just consider the off‐switching time for 488 nm illumination power of 0.25 kW/cm^2^, Dronpa2 and rsEGFP will return to the off‐state within 1.5 and 2.3 ms, respectively, 34 ms for both SkyLan‐NS and GMars‐Q, while rsEGFP(N205S) needs 9.4 ms (Figure 1c,d). Those values correspond to the time needed by the protein to reach 5% of the initial fluorescence of the photoswitchable fraction. The background fluorescence reached at the end of the off‐switching curves is the result of the on‐switching elicited by the 488 nm, and it defines the on/off contrast (Figure 1e).

The on‐switching rate, i.e., the off‐to‐on transition, is similar for the different RSFPs, except for GMars‐Q for which it appears to be slower (Figure 1f). What differs in the on‐transition is the fraction of RSFPs that can be actively switched between the two states [23, 24]. For rsEGFP2, around 20% of the initial fluorescence cannot be recovered, while for GMars‐Q and rsGreenF, more proteins than the ones in the on‐state at the thermal equilibrium can be switched on under the same illumination and pH conditions (Figure S1). This will affect the total number of visible fluorophores, an aspect to be considered when quantitative information needs to be inferred from the imaging.

To achieve high spatial resolution images as well as extended timelapse imaging, the RSFPs must sustain a large number of switching cycles before irreversibly losing fluorescence. Approximately 10 cycles are needed to increase the resolution of one order of magnitude per dimension; therefore, a 2D image would require at least 100 cycles. Since it implies less light dose per cycle, fast off‐switching kinetics are generally associated to an increased number of cycles, i.e., higher fatigue resistance. A comparison between the different RSFPs for the same on‐switching light intensities and the same duration of off‐illumination (enough to switch off 80% of the photoswitchable fraction) shows how Dronpa2 and rsEGFP2 are able to withstand more than 2000 cycles before losing more than 30%–40% of the initial fluorescence signal, while the slow RSFPs loose around 60%–70% of their fluorescence (Figure 1g). The weak fatigue resistance of SkyLan‐NS, sustaining less than 250 cycles before decreasing to half the fluorescence, severely impairs its use in prolonged RESOLFT recordings. Fatigue resistance depends on multiple parameters of imaging, like duration, intensity and wavelength of the illumination light [24].

### 2.2 RESOLFT Image Formation: From Experiment to Simulation

**FIGURE 2:**
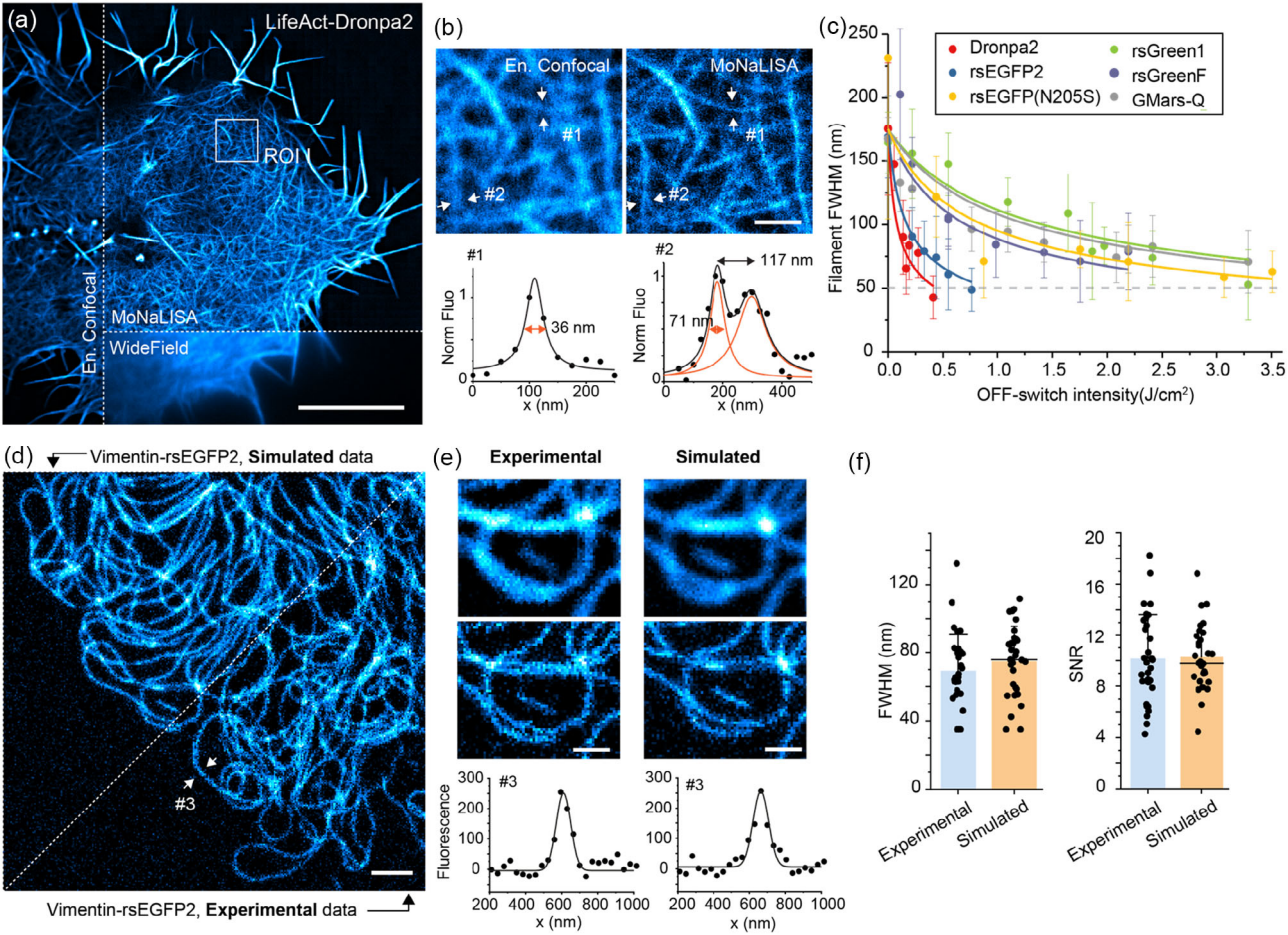
RESOLFT imaging for different RSFPs experimental and simulated. (a) Representative image of U2OS expressing LifeAct‐Dronpa2 comparing Widefield, enhanced confocal and parallelised RESOLFT/MoNaLISA. Scale bar, 10 µm. (b) Zoom‐in on ROI I, where the FWHM of different actin filaments is estimated through a Lorentzian fit, as well as the distance between two close‐by filaments. Scale bar, 1 µm. (c) Depletion curves for the array of green negative RSFPs calculated by estimating the FWHM of filamentous structure (vimentin or map2) at increasing off‐switching power. Each data point is the mean ± std of the FWHM for >30 frames over a 50 × 50 µm^2^ field of view modelled as a Lorentzian peak. (d) Representative comparison of experimental and simulated RESOLFT/MoNaLISA image of vimentin tagged with rsEGFP2. Scale bar, 1 µm. (e) Zoom‐in showing RESOLFT/MoNaLISA and enhanced confocal, together with line profile across one filament. Scale bar, 500 nm. (f) FWHM and SNR for experimental and simulated images of 25–30 line profiles across single filaments.

Most of the RSFPs characterised herein have been successfully used for RESOLFT imaging, as shown in Figure 2 for the parallelised RESOLFT techniques named MoNaLISA (Molecular Nanoscale Live Imaging with Sectioning Ability [12, 25]), resolving spatial features down to 40–70 nm at optimal illumination energy settings (Figure S2, S3 and Table S1 for the correlation between photophysical parameters and imaging condition). However, the choice of the illumination parameters influences the final image contrast and spatial resolution as shown in Figure 2c (and Figure S3) where the resolution is measured at increased off‐switching time.

Experimentally tracking the effect that each step of the image formation and RSFPs plays on the final image quality is challenging; many factors including cellular variability limit our confidence in probing resolution (Figure 2c and Figure S3). This is especially true for RESOLFT imaging where the live cell condition (i.e., no fixation methods and aqueous buffers) is essential to maintain an unperturbed and optimal on/off switching behaviour [26].

To enable a systematic exploration of the parameter space, we have built an open‐source simulation software based on the theoretical derivation of RESOLFT image formation [13]. Fluorophore switching is described as a two‐state Markov model fully described by the on‐to‐off and off‐to‐on cross section combined with the emission from the on‐state (Note S1). Although some fluorophores can exhibit characteristics not fully covered by this simplified model [24], it captures the general behaviour of RSFPs in the regime of low to moderate illumination intensities that distinguishes RESOLFT imaging [23], especially in its parallelised implementations. It also provides a framework for describing the behaviour of fluorophores using a compact set of parameters, facilitating both standardised measurement procedures to characterise fluorophores and a clean and comprehensible image formation model. We have extracted the on/off cross sections from the previous photophysical characterisation and used to model the photoswitching of the fluorophores and compare them (Note S2). From the same model, we derive the FRC curve to quantify information across spatial frequencies, and we report a single resolution value (*d*_FRC_) using the standard 1/7 threshold while comparing images only for the same fluorophore distribution to avoid sample dependence [27] (Note S3).

### 2.3 Influence of the Spatial and Temporal Illumination Sequence

The quality of the light patterns and the energy delivered during the illumination affect the resulting RESOLFT image quality. To investigate a given parameter, we keep all other parameters constant and calculate the predicted FRC resolution, *d*_FRC_, across a range of settings. Additionally, we provide simulated images of the same filamentous structure for different values of the parameter of interest to give visual examples. In all predictions and simulation in this section, we mimic rsEGFP2, commonly used in RESOLFT imaging. The simulation can generate images comparable to the one recorded experimentally as quantified in Figure 2d–f through FWHM of line profile across single filaments and signal‐to‐noise level across the image.

**FIGURE 3:**
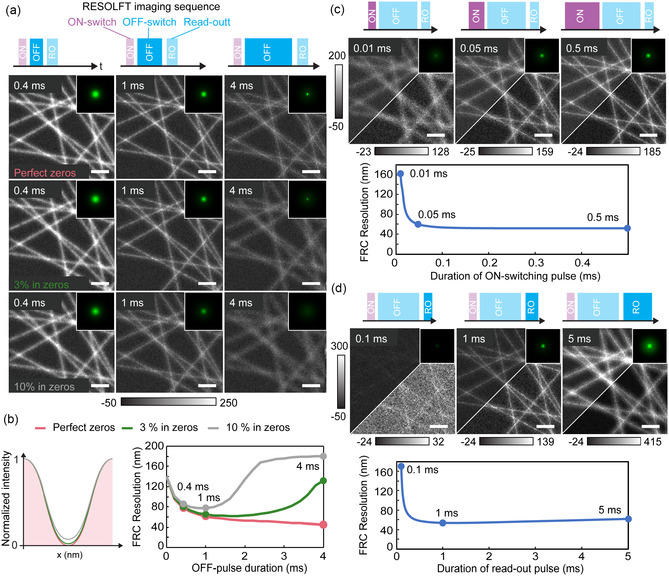
RESOLFT imaging dependence on the on–off illumination energy. The simulations are performed considering a rsEGFP2‐like photophysical parameter under a RESOLFT pulse sequence composed of on‐switching, off‐switching and read‐out. (a) Variation of both quality of the zero of the doughnut pattern and energy (in this case, by increasing the time of illumination at constant power) of the off‐illumination pulse. Three examples are reported for each explored parameter as both simulated images of filaments and kernel (inset). (b) Intensity profile of a doughnut with perfect 0% vs 3% or 10% residual intensity in the zero together with the graphs plotting FRC resolution as a function of read‐out pulse duration for the different zero qualities. (c) Variation of the on‐switching pulse duration. (d) Variation of the read‐out pulse duration. The simulation is performed considering a rsEGFP2‐like photophysical parameter under a RESOLFT pulse sequence composed of on‐switching, off‐switching and read‐out. Scale bars of each simulated image, 500 nm and kernel image of 540 µm per side. Each image is presented both at fixed count range across the screened parameter (left triangle) or adapting it to each condition individually (right triangle).

Firstly, we explore how different pulse durations during off‐switching illumination (Figure 3a,b) affect RESOLFT imaging. Longer off‐switching would provide a narrower effective point spread function and therefore images with higher resolution, but this is only true for the ideal case of perfect light pattern. Achieving a true zero is crucial for both RESOLFT and STED imaging, yet it is often difficult in practice due to imperfect interference or aberrations that introduce a small illumination dose at the expected zero. We simulate images for three illumination conditions defined by the residual intensity at the minimum relative to the crest of the doughnut. With perfect zeroes, the resolution follows the predicted diffraction unlimited behaviour. In the 3% and 10% cases, the resolution reaches a minimum and then increases because the central on state population is increasingly switched off. A 3% residual central intensity is realistic for a RESOLFT microscope [11, 12]. Therefore, in an experimental setting, an excess off‐pulse duration should be avoided as it not only increases the recording time but will also deteriorate the final image quality.

On‐switching illumination and read‐out also contribute to the resulting RESOLFT image quality as shown in Figure 3c,d. Firstly, the image quality mirrors the on‐switching profile; a sufficiently long pulse is essential to obtain signal, after which performance plateaus when the RSFPs are fully in the on state. It should also be noted here that many RSFPs need 405 nm light for on‐switch. This wavelength can also cause photoinduced fatigue which decreases the average number of switching cycles.

For the read‐out pulse, image quality improves as the pulse length increases up to approximately the off‐switching time, which is about 1 ms for the simulated rsEGFP2 conditions, and then deteriorates for longer read‐out times. Continued illumination causes the on‐state population in the confined region to switch off and equilibrate with its surroundings due to the on‐switching crosstalk from 488 nm illumination. The emitted photons then carry less information and begin to degrade, rather than improve, image quality. The extent of this effect depends on the fluorophore properties and should be considered carefully when selecting imaging parameters.

### 2.4 RSFPs Photon Rate and On/Off Contrast

**FIGURE 4:**
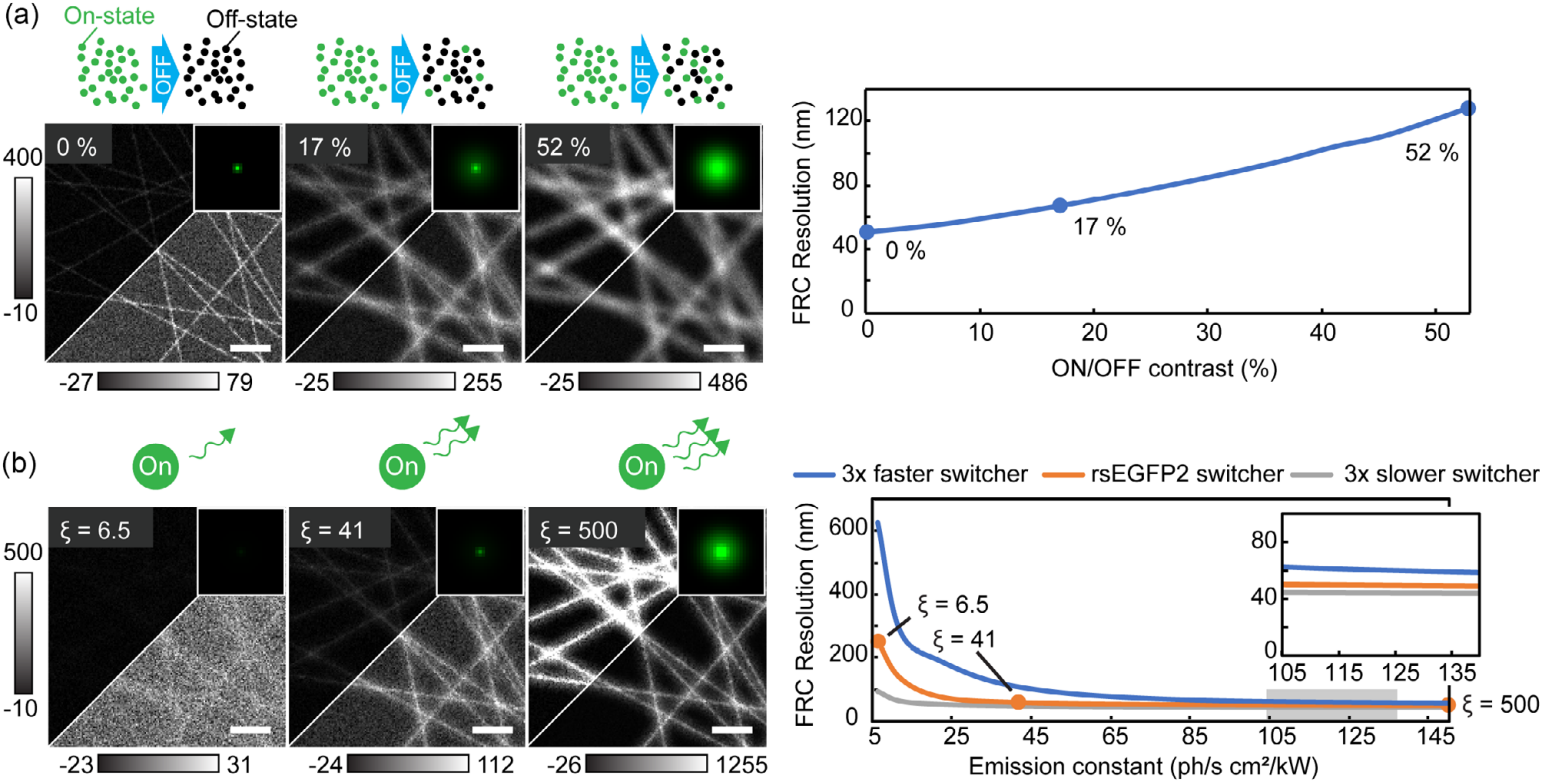
RESOLFT imaging dependence on negative rsFPs’ properties. The simulation starts from a rsEGFP2‐like set of parameters to selectively change its intrinsic properties in a rational way and compare them. (a) Imaging quality for increasing on/off switching contrast. (b) RESOLFT imaging with fluorophores having three different switching speeds, keeping the quality of off‐switching constant and adjusting off‐switching energy to keep final confinement constant. We then vary the emission constant. Scale bars of each simulated image, 500 nm and kernel image of 540 µm per side.

Besides the rate of on/off switching, another parameter that uniquely identifies RSFPs is the on/off contrast. It reports on the residual fluorescence reached upon continuous off‐switching illumination as a result of the crosstalk between on and off switching at a given wavelength. In Figure 4a, we track the change in image quality for an on/off contrast between 0% and 52%. The green expectation kernels and simulated images show a progressively higher confocal‐like background surrounding the centrally confined emission, which deteriorate the final image quality. Correspondingly, the *d*_FRC_ increases from below 50 nm at 0% incomplete switching up to above 120 nm at 52% incomplete switching. To generate fair comparisons, the off‐switching energy and the read‐out time were optimised for each individual point in the curve. Lower on/off contrast requires less off‐switching energy and shorter read‐out time to produce optimal image quality.

Additionally, we investigate the effect of the rate of fluorescence generated by the fluorophores in the on‐state when illuminated with the read‐out pulse. In Figure 4b, we show simulated images of structures labelled with RSFPs featuring three different levels of emission constant, defined as the photon rate over the illumination power density, and equal switching rates mimicking rsEGFP2. We extend this exploration reporting in the predicted *d*_FRC_ also at faster and slower off‐switching kinetics. Low emission constant translates in low image quality, but slower RSFPs mitigate the loss by allowing longer integration and therefore recovering signal. When the emission constant is increased, the image quality plateaus. The slower the RSFPs, the earlier this happens.

The plateauing behaviour stems from the fundamental property of a Poisson random variable where the SNR increases as the square root of the expectation value. For RSFPs and RESOLFT imaging, however, the level of the plateauing effect is increased by the existence of photoswitching noise [13]. Since the fluorophores switch between two states stochastically, and we can only manipulate the probability of the different states with light, there is an intrinsic uncertainty in the number of fluorophores in the on‐ and off‐state respectively. This propagates to the final image as pixel‐to‐pixel noise for point scanning systems and can in many cases be a stronger noise component than the more well‐known Poisson‐distributed photon shot noise [28]. As the fluorophore emission rate increases, the relative photon shot noise decreases and photoswitching noise becomes more dominant. Since the photoswitching noise is constant, a plateau is reached where photoswitching noise is the predominant noise source.

### 2.5 Positively Switching Fluorophores

**FIGURE 5:**
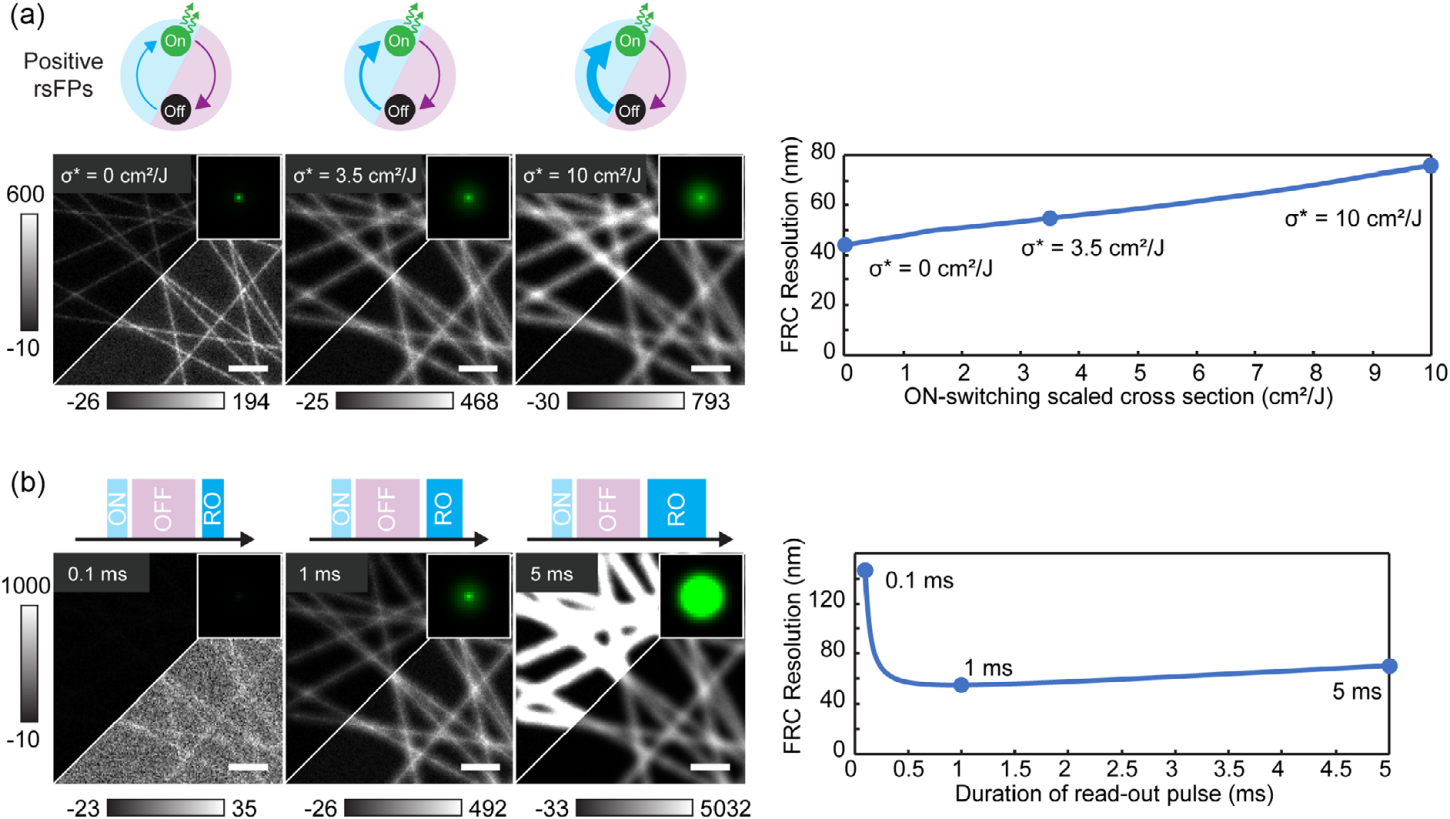
RESOLFT imaging dependence on positive RSFPs. The positive switcher is simulated inverting the kinetic rates of rsEGFP2‐like. (a) Change in the on‐switching cross section, i.e., how fast the fluorophores switch on during read‐out. In the figure, the cross section scaled by the energy is reported to remove the dependence to the laser power. (b) Fixing the on‐switching scaled cross section to 5.32 cm^2^/J, the length of the read‐out pulse is varied. Scale bars of each simulated image, 500 nm and kernel image of 540 nm per side.

So far, we have limited ourselves to varying parameters of negatively switching fluorophores, where the fluorescence excitation and off‐switching are coupled, meaning that the off‐switching and the read‐out pulses first generate fluorescence photons and then turn the RSFPs into the dark state [29]. In Figure 5, we instead look at positively switching fluorophores, for which the light in the read‐out pulse not only generates fluorescence but also switches more RSFPs into the on‐state. To enable a direct comparison within the same modelling framework, we present a theoretical analysis in which the observed complex photophysics of positive RSFPs [29, 30] is approximated by the same two‐state on/off switching model. As this section is not based on protein‐specific photophysical characterisation, the results are not directly tied to a specific positive RSFP. In Figure 5a, we vary the on‐switching cross section induced by the read‐out pulse, keeping the prior confinement constant. Large on‐switching cross section gives a deterioration of the expectation kernel and thus the final image. Notable is that as the on‐switching cross section tends to zero, the switching can be said to be decoupled, as the read‐out pulse neither turns the fluorophores on or off. In Figure 5b, we fix the on‐switching cross section induced by the read‐out pulse and vary the duration of the read‐out pulse. Also in this case, there is a clear optimal read‐out time, corresponding to a balance between sufficient photon emission within the focal region while minimising peripheral on switching. The deterioration in image quality for longer read‐out times appears to be more severe for positive switchers than for negative switchers.

### 2.6 Switching Fatigue

In the analysis so far, we have worked under the assumption that the fluorophore distribution is constant and stationary throughout the acquisition. Considering the number of cycles sustained by commonly used negative switcher such as rsEGFP2 or Dronpa2, this assumption is valid during the acquisition of a single RESOLFT image. However, when extending the observation to multiple time points, the finite number of switching cycle [19, 24, 31] per protein is what limit the accessible time window by progressively deteriorating image quality.

**FIGURE 6:**
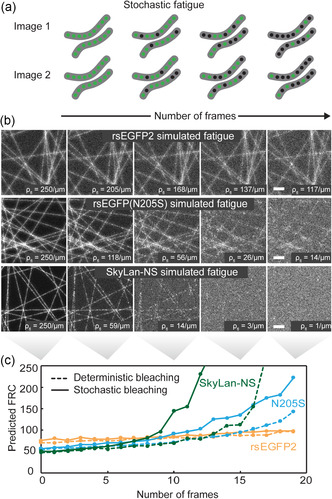
Photoswitching fatigue influence on RESOLFT imaging. (a) Schematic illustration of the fatigue stochasticity, which induces labelling noise. (b) Images of filamentous structures with decreasing labelling density. Image sequences are simulated with three different fluorophore properties that are intended to mimic the observed behaviour of rsEGFP2, rsEGFP(N205S) and SkyLan‐NS. (c) Same structure with different estimation of the FRC either deterministic, i.e., using the same image for the FRC calculation (dashed lines) or stochastic, i.e., using two images containing a different subset of fluorophore as a consequence of fatigue (solid lines). Three different proteins characterised by different off‐switching speed and emission constant are simulated. Scale bars of each simulated image, 500 nm and kernel image of 540 µm per side.

From an image formation perspective, fluorophores that have undergone irreversible photobleaching can be removed from the model, effectively translating in a decrease in labelling density. A sparser fluorophore distribution will have lower spectral power, essentially meaning there will be less total signal to image. If imaged with the same parameters, decreasing spectral power of the sample will result in worse image quality. In Figure 6, we report images of the same type of filamentous structure but with exponentially decreasing fluorophore density to simulate the fatigue process. The degradation point is detectable earlier by visual inspection than by the FRC curve as a result of the loss in correlation between the fluorophore distribution and the underlying structure. We here refer to this as labelling noise. Let us consider a filamentous structure with a density of 250 fluorophores per micrometre. At the beginning of the imaging, before significant fluorophore fatigue has occurred, the structure will be evenly labelled. The correlation between the biological sample and the fluorophore density is high. After imaging the sample for a long time and thus inducing fatigue, the number of fluorophores along the filaments will decrease as the fluorophores stochastically undergo photobleaching. Part of the filament may still contain many functioning fluorophores while others contain none, even though the underlying filament is homogenous in nature. The correlation between the sample and the fluorophore density has weakened due to labelling noise (Figure 6).

Labelling noise is not accounted for in our image formation model. To investigate whether labelling noise contributes significantly to image degradation of fatiguing samples, we devised an experiment to test this. Here, instead of deterministically removing fluorophores to mimic fluorophore fatigue, we create replicas of the same underlying filamentous structure but with stochastically sampled labelling at the corresponding fatigue conditions. We then simulate images of the fatiguing samples and measure the experimental *d*_FRC_ values at the different timepoints. These *d*_FRC_ values will thus incorporate both the effect of decreasing spectral power and the increasing labelling noise that comes with fluorophore fatigue. As a comparison, we do the exact same experiment but with deterministically decreasing number of labels. The results show that for labels mimicking rsEGFP2, the effect of labelling noise is negligible due to the high fatigue resistance of the RSFP. For weaker fluorophores, like rsEGFP(N205S) and SkyLan‐NS, labelling noise does play a significant role as the *d*_FRC_ of the images with and without labelling noise clearly diverge. The reason that labelling noise is more significant for rsEGFP(N205S) and SkyLan‐NS is their slower switching speed and therefore higher emitted photons per cycle. This means that the fluorophore distribution can be accurately imaged even at low labelling densities. On the other hand, with faster proteins, such as rsEGFP2, a denser labelling is required to achieve a good image of the fluorophore distribution.

## 3 Material and Methods

### 3.1 Cell Culture

U2OS (ATCC HTB‐96) cells were cultured in Dulbecco's modified Eagle medium (DMEM) (Thermo Fisher Scientific, 41966029) supplemented with 10% (vol/vol) fetal bovine serum (Thermo Fisher Scientific, 10270106) and 1% penicillin–streptomycin (Sigma Aldrich, P4333) and maintained at 37°C and 5% CO_2_ in a humidified incubator. For transfection, 1 × 10^5^ cells per well were seeded on 18 mm coverslips. After 1 day, cells were transfected using Fugene according to the manufacturer's instructions. Thirty‐six to forty‐eight hours after transfection, cells were washed in phosphate‐buffered saline (PBS) solution, placed in phenol red‐free DMEM or Leibovitz's L‐15 medium (Thermo Fisher Scientific, 21083027) in a chamber and imaged at room temperature.

### 3.2 Comparative Photophysical Characterisation

To record the photophysical behaviour of the different RSFPs on the same platform, we used a wide‐field illumination (obtained removing the microlenses from the MoNaLISA setup [12]) for the 488 nm excitation and 405 nm on‐switching laser, with a FWHM of the beam around 50 µm for both the wavelengths. To avoid the convolution of different powers along the Gaussian profile of the beam and to drive the camera at the fastest speed, an area of 31 × 1 µm^2^ at the centre of the light distribution is considered for the analysis. The off‐switching kinetics is recorded in a pump‐probe modality, where a 1 ms pulse of 405 nm light (at 0.09 kW/cm^2^) is followed by a 488 nm pulse for the time needed to switch the molecule completely off or at least to a stable residual fluorescence level. The cycle is repeated 25 times, and the curve is presented as mean ± std. The off time extracted from the curve is the 1/e decay time. The plateau level reached by the fluorescence at the end of the 488 nm light pulse is reported as the background level. The on‐switching has been investigated maintaining the same power of excitation (118 W/cm^2^) and gradually increasing the power of 405 nm used to switch on the same portion of the cell for 1 ms. The excitation with 488 nm light is kept for the time needed to switch the protein back off at 80% of the photoswitchable fraction. The curves are shown as mean ± std of three repetitions in different cells. The power dependency has been reported either normalised to the level of fluorescence before any 405 nm illumination, to highlight the resting state of the protein, or to the maximum value, to highlight the speed of the process. The fatigue curves describe the number of cycles sustained by each protein given the same on‐ and off‐switching powers (405 nm at 66 W/cm^2^ and 488 nm at 262 W/cm^2^). After 1 ms of on‐switching light, the protein is illuminated with blue light (off‐switching) for the time needed to have an 80% decrease of the initial fluorescence of the photoswitchable fraction (1.6 ms for rsEGFP2, 1 ms for Dronpa2, 6.3 ms for rsEGFP, 3.4 ms for rsGreen1, 2.5 ms for rsGreenF, 17 ms for GMars and 19.5 ms for SkyLan‐NS). Given the on‐switching power, the process is not saturated but is switching on a fraction of fluorescent protein between 50% and 80% of the total underlying concentration. The measurements are conducted in cell, and each curve is the mean ± std of three to five repetitions in different cells. The constructs used for the fatigue analysis are vimentin‐rsEGFP2, LifeAct‐Dronpa2, Map2‐rsEGFP, Map‐rsGreen1, Map‐rsGreenF, LifeAct‐GMars and LifeAct‐SkyLan‐NS.

### 3.3 Simulation Software

The simulation software is based on the theoretical framework developed in Boden et al. [13]. The software is developed in Python (≥3.7) and relies on NumPy and SciPy for numerical computation, with additional dependencies for image handling and processing (scikit‐image, imagecodecs), just‐in‐time acceleration (numba) and the graphical user interface (PyQt5, pyqtgraph, PySignal); the full list of required packages and version constraints is provided in the repository's requirements.

The simulation software implements a forward model of RESOLFT image formation using a two‐state (on/off) switching scheme. For a given fluorophore parameter set, illumination sequence and optical/detection configuration, it predicts the expected image and corresponding noise statistics through the computation of expectation and variance kernels. Each input parameter can be specifically defined or investigated over a range to directly visualise changes of image quality over the specified range. The full set of parameters can be saved in a configuration file (JSON format). From these kernels, the software predicts the FRC curve and extracts a single resolution metric (*d*_FRC_) using the 1/7 threshold; unless stated otherwise, FRC predictions are calculated assuming a spectrally flat sample for consistent comparisons across conditions. Representative simulated images are generated by adding pixel‐wise random noise consistent with the predicted variance and can be exported as output (TIF format) of the simulation together with the FRC prediction and resulting kernels.

## 4 Discussion

In this work, we present a photophysical characterisation, a minimalist theoretical framework and a free open‐source software for investigating the link between imaging parameters and image quality in the general framework of RESOLFT nanoscopy, allowing for an objective and quantitative optimisation of image parameters. Additionally, the work provides insights into how fluorophore properties affect image quality which can guide the choice of fluorophores for given applications as well as provide valuable information for the development of new fluorophores. Both representative images and FRC are complementary used as a comparative metrics to evaluate image quality. Although the absolute values acquired from the *d*_FRC_ threshold are not always indicative of image quality, we show that the relative changes in *d*_FRC_ values when imaging the same sample reflect the changes in image quality in terms of the ability to quantify small spatial features.

We analyse several parameters relating to both the imaging system and the properties of the fluorophore. From the results, we see that for many of the parameters explored, there are local optimal values from where both a decrease and an increase will deteriorate the image. For some parameters, like the off‐switching time with a non‐perfect doughnut, the local optimum is narrow and even a slight deviation from it will severely harm image quality. For others, like the read‐out time, using a too large value will not have such a detrimental effect, whereas a too small value will give a much worse image quality. The parameters of the fluorophores also affect the image quality in a non‐trivial way, and different fluorophores may require different imaging parameters to maximise the performance. These results in many aspects aligned with our practical experiences from RESOLFT imaging and confirmed the importance of correct parameters. The results presented here often entail fixing all but one of the parameters and examining the effect of that last one. It should be emphasised that this effect is often dependent on the values of the fixed parameters and should not be taken as a fully generalised effect.

Fluorophore fatigue is an often‐encountered challenge in RESOLFT timelapse imaging; we see from measurements on simulated data that image deterioration from fatigue can be due to both a decrease of the sample spectral power and from the stochasticity of the fatigue process. For faster switching fluorophores like rsEGFP2, fatigue‐related image deterioration is primarily due to decreased spectral power which increases the effect of photoswitching and Poisson noise. For slower switchers rsEGFP(N205S) and SkyLan‐NS, the deterioration is also highly influenced by labelling noise. By generating virtual samples including labelling noise and simulating images of these samples, we can also explore this effect and illustrate the trade‐offs between the choice of RSFP and imaging parameters to achieve the required image quality over multiple frames.

The full range of parameters to potentially explore is much larger, and the parameters of interest may differ vastly between applications and RSFPs investigated. We therefore provide the prediction software used as an open‐source project giving anyone the possibility to explore their parameters of choice. The software provides both FRC curve predictions and simulated images of virtual samples. The user may set parameters relating to the fluorophore, the pulse scheme, the optical system, the scanning step size, sample properties and the detector type. The software also allows the user to set ranges for parameters to easily find and optimise certain parameters within a given range. Parameters are easily saved and loaded, and results can be saved for analysis or visualisation outside the software.

The framework presented here deals with the image formation process of RESOLFT point scanning systems and treats only the case of flat (2D) samples. For cases where there is no significant crosstalk between adjacent foci, it can also be considered valid for parallelised point scanning systems, such as MoNaLISA [12] imaging and 3DpRESOLFT [32]. An extension of the image formation model to also consider the 3D nature of samples would be conceptually straightforward but would add complexity to the interpretation of the results and is thus left as a potential next step in this line of work. Equally important may be developing more sophisticated models of fluorophore behaviour to capture more subtle differences between reversibly switchable fluorophores such as multiexponential off‐switching and complex fatigue profiles. Further development in this direction will help generate even more accurate predictions of image quality.

### 4.1 Scope and Limitations

The software is intended as a practical bridge from measured photophysics to RESOLFT image formation: it allows users to explore how RSFP properties (e.g., brightness/emission rate, switching cross sections, on/off contrast, fatigue resistance), sample parameters (e.g., labelling density) and illumination sequences jointly determine predicted image quality and to decompose the contribution of each parameter to support imaging scheme selection and fluorescent probe or microscope design. FRC‐based outputs are best interpreted as relative trends under controlled comparisons rather than an absolute measure of achievable resolution. Its predictions rely on a minimalist two‐state (on/off) switching model and therefore do not capture more complex photocycle features, such as additional transient states, thermal recovery or cycle‐to‐cycle changes. In particular, the current implementation does not account for photoswitching fatigue dynamics during the acquisition of a single frame or across timelapse sequences. Sample and image formation is currently limited in 2D (2D structures and PSF/RESOLFT kernels) and does not include spatially local dependency that can modulate photophysics in cells.

## Supporting Information

Additional supporting information can be found online in the Supporting Information section.

**Supporting Fig. 1**: Fraction of protein that can be on‐switched after the first cycle. The data derives from the same dataset of the one reported in Figure 1f but shows the ratio between the fluorescence before any illumination with 405 nm light and after reaching the plateau at increasing illumination energy. **Supporting Fig. 2**: Examples of RESOLFT imaging for different RSFPs tagged to cytoskeleton proteins at optimal imaging conditions (i.e., the parameter reported in Table S1). **Supporting Fig. 3**: Depletion curves and examples for different RSFPs. (a) Examples from four off switch at progressively higher energy of 488 nm off‐switching light. Scale bar, 1 μm. FWHM (b) and signal‐to‐noise ratio (c) as a function of the off‐switch energy. The experimental data points are plotted together with the fitted resolution curves and the average value over the data respectively. Each data point is the mean ± std of the FWHM for >30 frames over a 50 × 50 μm^2^ field of view modelled as a Lorentzian peak. **Supporting Fig. 4**: Software forFRC prediction and image simulation. (a) The software developed takes as input the switching parameters of the RSFPs, the illumination scheme and the properties of the optical and computational detection. (b) The graphical user interface allows for easy access to all the input parameters using numerical inputs and graphical aids. The results section on the right displays the output as graphs and images and allows the user to readily scan through the input range of parameters. **Supporting Fig. 5**: Impact of sample density on FRC values. FRC curves reflect how much signal is present in an image of a structure at different frequencies with respect to the noise at that frequency. The value not only depends on the properties of the image formation process, but also inherently on how much of the given frequency is presented in the underlying fluorophore density. If there is no or very little energy in the fluorophore density at a certain frequency, there will surely not be much useful signal in the image at that same frequency. We illustrate this by simulating images of three samples composed by virtual DNA origami structures consisting of two pools of 20 fluorophores each separated by 100 nm. The different samples have (a) 10, (b) 100 and (c) 1000 replicas of these DNA origami structures each in their field of view. The three origami samples roughly have equally shaped spectral profile, although with increasing spectral energy more origami structures are present. Thus, although the imaging system is exactly the same, the FRC curve will differ vastly and so also the resulting FRC resolution value as defined by the 1/7 threshold. In all three images though, we will claim that the two fluorophore pools can be readily, and equally well, observed as separate points in the three images. The upper row shows the full images. The lower row shows the experimentally measured FRC curves in blue along with the predicted FRC curves for a spectrally flat sample with the same total energy as the imaged sample in green. Yellow dashed line shows the 1/7 threshold. Inset shows zooms of highlighted regions along with line profiles on top as indicated by the white arrows. **Supporting Fig. 6**: FRC curves predicts the increase in resolving power. We generate virtual samples consisting of simple structures distributed on a uniform grid. We simulate images of the sample using read‐out pulses of different length. Using straightforward and simple quantification algorithms, we quantify properties of the underlying sample from raw and deconvolved images. (a) Shows the full images of the virtual samples together with an inset of one of the sub‐regions emphasising the angle α between the lines and the radius r of the circle. (b) Shows the same sub‐regions as in (a) imaged with our simulation tool at different durations of the read‐out pulse. Top rows show raw data and bottom rows show deconvolved data. (c) Shows standard deviation and bias of the estimations for the raw and deconvolved data at different read‐out pulse durations together with the predicted FRC values for the different durations. **Supporting Fig. 7**: Characterisation of the switching cross section for rsEGFP2. (a) Off‐switching decay at increasing intensity of 488 nm light, fitted with an exponential decay with rates following a gamma distribution. Each curve is the mean ± std of at least 3 independent measurements on different cells. (b) Power dependency of the off‐switching rates (error estimated from the fitted model) and linear fit. (c) Background, i.e., residual fluorescence after prolonged 488 nm light illumination, mean ± std of the last 50 points of the curve in (a). (d) On‐switching curve reporting the increase fluorescence recovered at increased intensity of the 405 nm light. The curve is normalised to the value of fluorescence of the first cycle. Each point is the mean ± std of at least 3 independent measurements on different cells. **Supporting Fig. 8**: Direct comparison of experimental (a) and simulated (b) RESOLFT images. The skeleton of the vimentin‐rsEGFP2 of panel a has been used as input image for the simulation in panel b. (c, d) Zoom‐in for both images in confocal and RESOLFT mode, with line profile across the region marked in the images (grey lines are experimental confocal, black dots RESOLFT data and solid black lines are fitted Lorentzian over the data). (e) Collection of the software parameters for the generation of panel b. **Supporting Fig. 9**: Image quality over time by FRC prediction for spectrally flat samples with an exponentially decreasing number of labels on each side. **Supporting Fig. 10**: Off‐switching kinetics for different cytoskeletal construct of GMars‐Q, Dronpa2 and rsEGFP(N205S). **Supporting Table 1**: Photophysical and imaging parameters for negative reversibly switchable fluorescent proteins reported in the study. The table collects the photophysical parameters according to the simple two‐state switching model assumed by the prediction software and the imaging parameters used for the collection of the images in Figure S2. The cross section for the on‐to‐off and off‐to‐on transition are estimated from the experimental data assuming for the 488 nm light both directions of switching and for the 405 nm only the off‐to‐on transition. The intensity of saturation is calculated by fitting the FWHM in function of the off‐switching illumination according to the formula $d=\lambda /2NA\sqrt{\left(1-I/I_{\mathrm{sat}}\right)}$. **Supporting Note 1**: Image formation model and Simulation software. **Supporting Note 2**: FRC as image quality metric. **Supporting Note 3**: From experimental parameters to image simulation.

## Funding

This study was supported by HORIZON EUROPE European Research Council (grant 101002490).

## Conflicts of Interest

The authors declare no conflicts of interest.

## Supporting information

Supplementary Material

## Data Availability

The data that support the findings of this study are available in the Zenodo repository 10.5281/zenodo.18144981. The image generator software used in this study is open source and available at https://github.com/TestaLab/Resolution_prediction_software.
