# Supplementary material for: A Versatile Tool to Predict and Guide RESOLFT Images Based on Photoswitching, Labelling and Optical Properties
Source: Chemphyschem. 2026 Apr 21;27(8):e202500780. doi: 10.1002/cphc.202500780 (PMC13099251; doi:10.1002/cphc.202500780)
Supplement: Supplementary file 1 — Supplementary Material [file CPHC-27-e202500780-s001.pdf]

Supplementary information for

A versatile tool to predict and validate RESOLFT images based on photoswitching, labelling, and optical properties

Boden et al.

Table 1. Photophysical and imaging parameters for negative reversibly switchable fluorescent proteins reported in the study. The table collects the photophysical parameters according to the simple two state switching model assumed by the prediction software and the imaging parameters used for the collection of the images in Figure 2. The cross section for the on-to-off and off-to-on transition are estimated from the experimental data assuming for the 488 nm light both directions of switching and for the 405 nm only the off-to-on transition. The intensity of saturation is calculated by fitting the FWHM in function of the OFF-switching illumination according to the formula $d = \lambda/2NA\sqrt{(1 - I/I_{sat})}$.

rsFPs	ON ↔ OFF Cross-sections			RESOLFT imaging parameters			I_{sat} [J/cm²]
	σ_{OFF}^{488} [×10⁻¹⁹ cm²] /QY_{OFF}^{488} [×10⁻²]	σ_{ON}^{488} [×10⁻¹⁹ cm²] /QY_{ON}^{488} [×10⁻⁴]	σ_{ON}^{405} [×10⁻¹⁸ cm²]	ON-switch (405 nm) [kW/cm² × ms]	OFF-switch (488 nm) [kW/cm² × ms]	Readout (488 nm) [kW/cm² × ms]	
**Dronpa2**	37 ± 1<br>1.6 ± 0.2	0.3 ± 0.1<br>1.3 ± 0.5	25 ± 2	0.6 × 0.5	0.41 × 1	1.3 × 1.5	17.7 ± 10
**rsEGFP2**	23 ± 1<br>1.0 ± 0.1	0.9 ± 0.1<br>3.9 ± 0.6	23 ± 2	0.6 × 0.5	0.41 × 1.5	1.3 × 1.5	12.6 ± 2
**rsEGFP**	4.30 ± 0.05<br>0.24 ± 0.01	0.24 ± 0.03<br>1.3 ± 0.2	28 ± 3	0.6 × 0.5	0.56 × 4	1.3 × 1.5	2.3 ± 0.7
**rsGreen1**	13.6 ± 0.2<br>0.62 ± 0.02	0.28 ± 0.08<br>1.3 ± 0.4	33 ± 3	0.6 × 0.5	0.561 × 4	1.3 × 1.5	1.5 ± 0.5
**rsGreenF**	29 ± 1<br>1.8 ± 0.1	0.7 ± 0.8<br>4.5 ± 5	14 ± 3	0.6 × 0.5	0.22 × 4	1.3 × 1	2.6 ± 0.9
**GMars-Q**	2.70 ± 0.05<br>0.18 ± 0.01	0.14 ± 0.01<br>1 ± 0.1	9 ± 4	0.6 × 0.5	0.561 × 4	1.3 × 1.5	2.3 ± 0.7
**SkyLan-NS**	4.7 ± 0.1<br>0.09 ± 0.004	0.09 ± 0.09<br>0.2 ± 0.1	26 ± 5	-	-	-	-

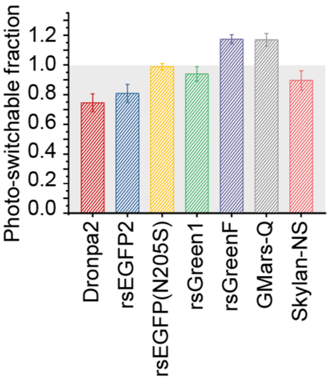

Supplementary Figure 1. Fraction of protein that can be on-switched after the first cycle. The data derives from the same dataset of the one reported in Figure 1f but shows the ratio between the fluorescence before any illumination with 405 nm light and after reaching the plateau at increasing illumination energy.

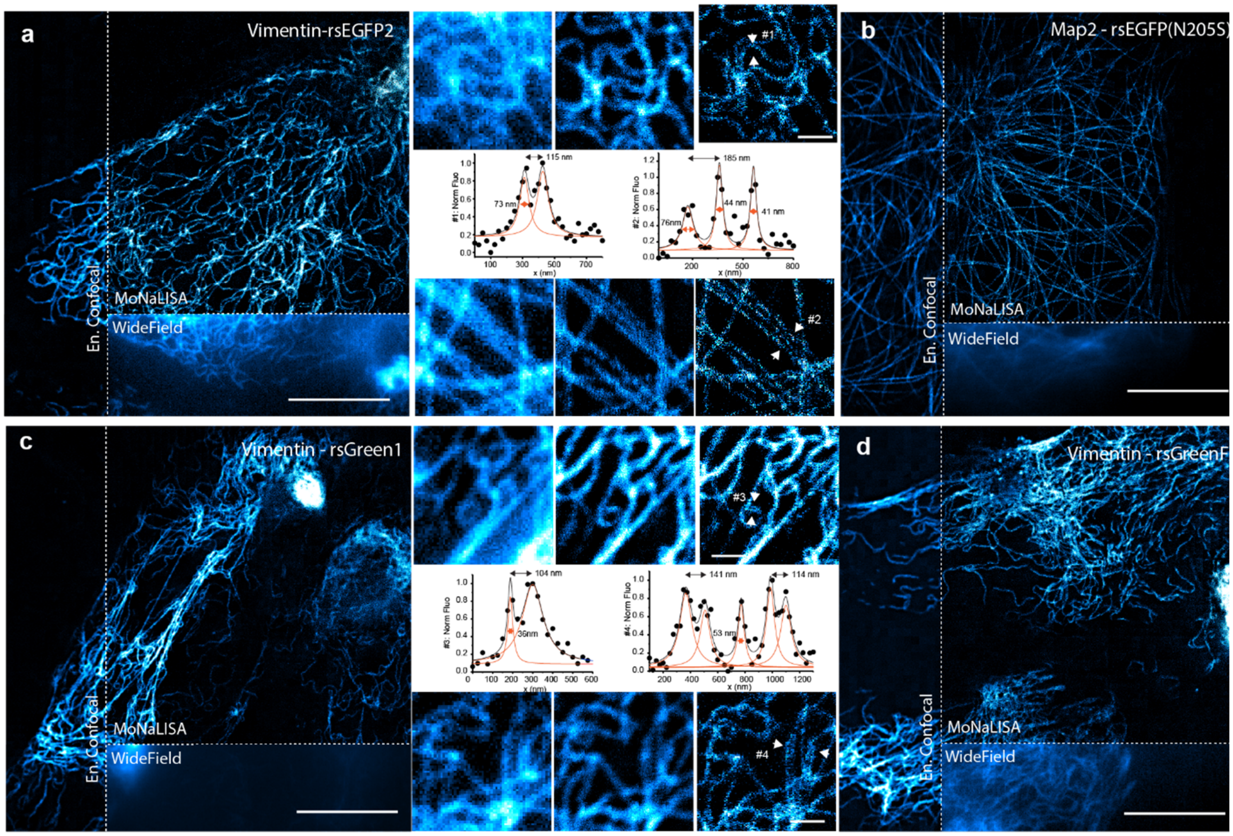

Supplementary Figure 2. Examples of RESOLFT imaging for different RSFPs tagged to cytoskeleton proteins at optimal imaging conditions (i.e. the parameter reported in Table 1).

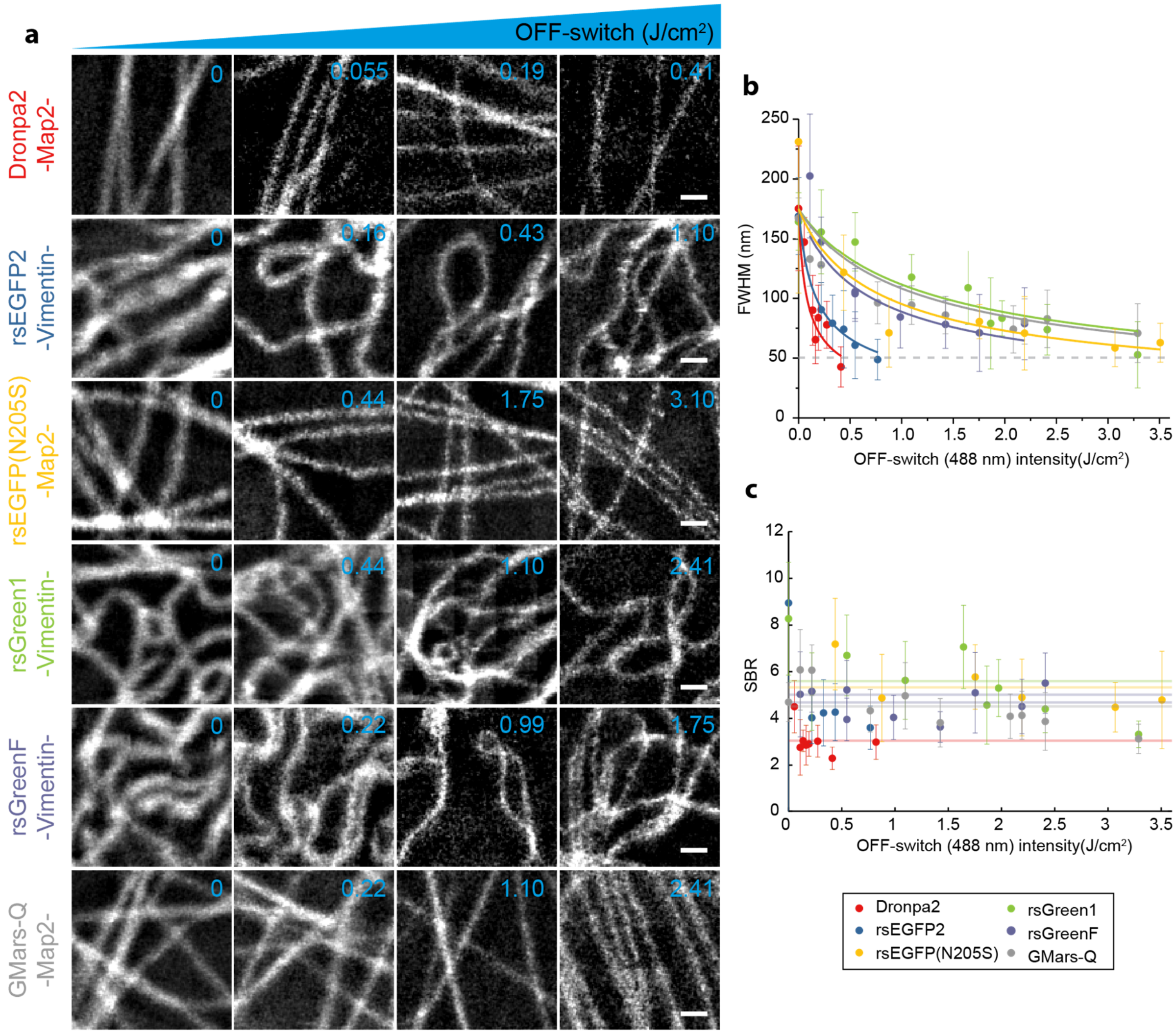

Supplementary Figure 3. Depletion curves and examples for different rsFPs. (a) Examples from four OFF-switch at progressively higher energy of 488 nm off-switching light. Scale bar, 1 µm. FWHM (b) and signal-to-noise ratio (c) as a function of the OFF-switch energy. The experimental data points are plotted togheter with the fitted resolution curves and the average value over the data respectively. Each data point is the mean ± std of the FWHM for >30 frames over a 50 × 50 µm^2 field-of-view modelled as a Lorentzian peak.

Supplementary Note 1. Image formation model and Simulation software

The pulse schemes used in point scanning RESOLFT microscopy involve creating a confined area of emitting RSFPs by switching on fluorophores with a focused illumination and then confining the area of fluorophores populating the on-state using a doughnut (or array of doughnuts) illumination pattern featuring a "zero" of intensity that switches off all fluorophores not located in the zero-intensity region of the illumination pattern. This confined area is then stimulated to emit by a third focused read-out illumination pulse. This combination of illumination pulses together generates fluorescence emitted primarily from a confined area centred on the coordinate defined by the scan position. Assuming the model of the fluorophores described above and with knowledge of the fluorophore properties, illumination patterns and their intensities, the exact emission from each point in the sample at a certain scan position can be predicted with its expectation value and variance[21]. Furthermore, also taking the detection system into account, the final image generated by the raster scan can be described with its pixel expectation and variance values as convolutions between the underlying fluorophore density and what we will henceforth refer to as the expectation and variance kernels.

$$E[Im[i,j]] = (d[m,n] * h_E[m,n])[i,j] \tag{1}$$

$$Var[Im[i,j]] = (d[m,n] * h_{Var}[m,n])[i,j] + \sigma^2 \tag{2}$$

where $Im[i,j]$ is the final image, $d[m,n]$ is the sample density, $h_E[m,n]$ and $h_{Var}[m,n]$ are the expectation and variance kernels and σ^2 is the variance induced by detector read-out noise. The expectation ($h_E[m,n]$) and variance kernels ($h_E[m,n]$) depend mainly on the imprinted spatial on-state distribution and the resulting spatial emission but are also influenced by the optical system properties as well as the quantification algorithm used. The full derivation of the kernels is shown in previous work[21].

Expressing the final image properties using these simple convolutions is the basis of our analysis, it allows us to readily predict the Fourier ring correlation (FRC) curves of the resulting images and simulate example images that well approximate what one would expect in a real system.

With knowledge of the expectation and variance kernels, predicting the FRC curves and simulating images according to the model described above is done using a software illustrated in Supp. Supplementary Figure . The FRC curve is calculated from the expectation and variance kernels H_E and H_{Var} of the imaging system together with the power spectrum ϕ_D and the average value $D[0,0]$ of the sample density as well the detector read-out variance σ^2.

$$FRC[r] = \frac{(|H_E[r]|^2\ \phi_D[r])}{|H_E[r]|^2\ \phi_D[r] + H_{Var}[0]D[0,0] + \sigma^2} \tag{3}$$

The FRC curve depends on the properties of the sample through the ϕ_D. To predict FRC curves in this work, we will assume that the sample being imaged is spectrally flat, meaning that the underlying structure contains equal signal power at every non-zero frequency components. We believe this makes for the most informative comparisons as the analysis is not weighted towards any certain spatial frequency. It is also reasonable to believe that most biological sample exhibit a

relatively flat power spectrum. To generate a simulated image, an expectation and a variance image is first generated by simply convolving the sample density with the corresponding kernels. The final image is then generated by adding a random value to each pixel in the expectation image with the variance determined by the corresponding value of the variance image. Since the resulting images are used as a visual aid, we settled with adding the noise as a normally distributed random variable. An exact probability density function for the noise distribution has not been derived.

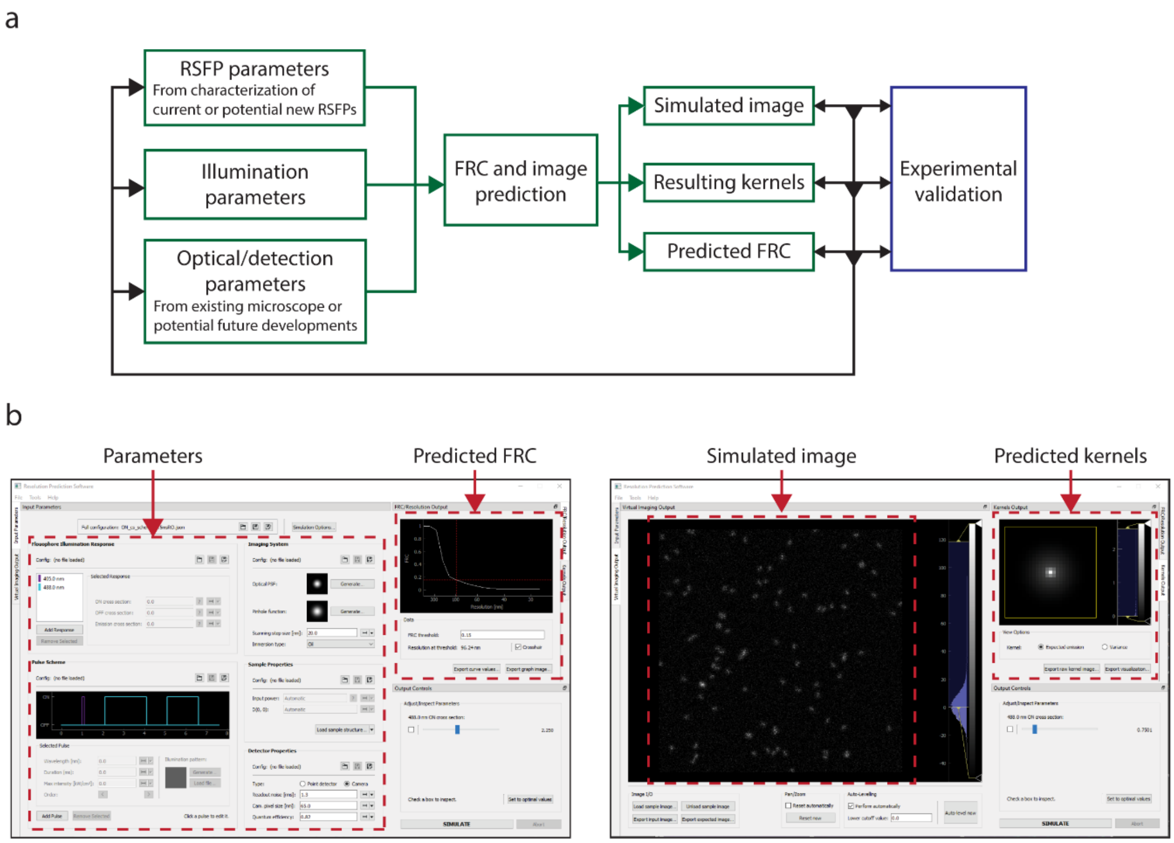

Supplementary Figure 4. Software for frc prediction and image simulation. (a) The software developed takes as input the switching parameters of the RSFPs, the illumination scheme, and the properties of the optical and computational detection. **(b)** The graphical user interface allows for easy access to all the input parameters using numerical inputs and graphical aids. The results section on the right displays the output as graphs and images and allows the user to readily scan through the input range of parameters.

Supplementary Note 2. FRC as image quality metric

FRC will be used to compare the performance of point scanning RESOLFT systems using different imaging parameters and different fluorophore properties. The FRC curve is directly derivable from the image formation model as shown in equation (3). FRC is a commonly used metric for describing or measuring the resolution of an imaging system. An experimentally measured FRC curve shows the correlation between two independently acquired images of the same underlying structure. High correlation implies a high signal-to-noise ratio, meaning large information content at the specific spatial frequency. As is common practice when using FRC[11] curves for resolution estimation, we will henceforth use the 1/7 threshold to extract a single resolution metric from the FRC curve, meaning that the resolution is defined as the frequency where the FRC curve falls under a value of 1/7. We will refer to this as the d_{FRC} value to distinguish it from the FRC curve. When using any metric as an estimate for resolution, it is essential to understand exactly what the metric measures in order to properly interprets its values. The FRC curve, as mentioned above, describes the information content at different spatial frequencies of the image. As is seen from equation (3), in order to have any information content in the image at a frequency corresponding to radius r, the underlying fluorophore density needs to have some, or sufficient, information at that frequency. In other words, $\phi_D[r]$ needs to be sufficiently large. Thus, it is important to acknowledge the tight dependence of the FRC curve, and therefore the d_{FRC} value, on the properties of the underlying sample. We illustrate this concept in Supp. Supplementary Figure where we simulate images of virtual samples imitating DNA origami structures where two pools of fluorophores are placed 100 nm apart. Three images with identical imaging parameters are simulated of samples with a different total number of origami structures in the field of view. Fewer origami structures mean lower spectral power over the whole spectrum, so even though the imaging system is constant and the apparent possibility to separate the two fluorophore pools within the structures is the same, the images give vastly different FRC curves and thus d_{FRC} values. For the purpose of our study however, this sample dependency can be overcome by only comparing images of the same underlying fluorophore distribution. For images of the same sample, we hypothesize that those with higher d_{FRC} values generally provide better information from which to draw conclusion about the underlying fluorophore distribution. To test this assumption, we compared the d_{FRC} values of images with the accuracy of a simple quantification algorithm. The results are shown in Supp. Supplementary figure . We generate a sample divided into a grid structure. In the first case, each sub-area of the grid contains two short filaments with randomly distributed fluorophores forming a cross with a random crossing angle. In the second case, each sub-area of the grid contains a circular area of random radius, labelled with randomly positioned fluorophores. Both samples are imaged with increasing duration of the read-out pulse resulting in different image quality. The images of the samples, both raw and deconvolved, are then fed into the quantification algorithm that finds the most likely cross angle or radii by simply generating sample structures will all possible angles and radii and identifies the strongest correlating one. The precision and the accuracy of the estimations are reported in the plots as the standard deviation and the bias of the estimations. The plots show that the precision of the estimations, in both cases, follows the trend of the d_{FRC} values showing minimum standard deviation at read-out times very similar to the ones giving the best d_{FRC} value. It can also be noted that deconvolution decreases the bias of the

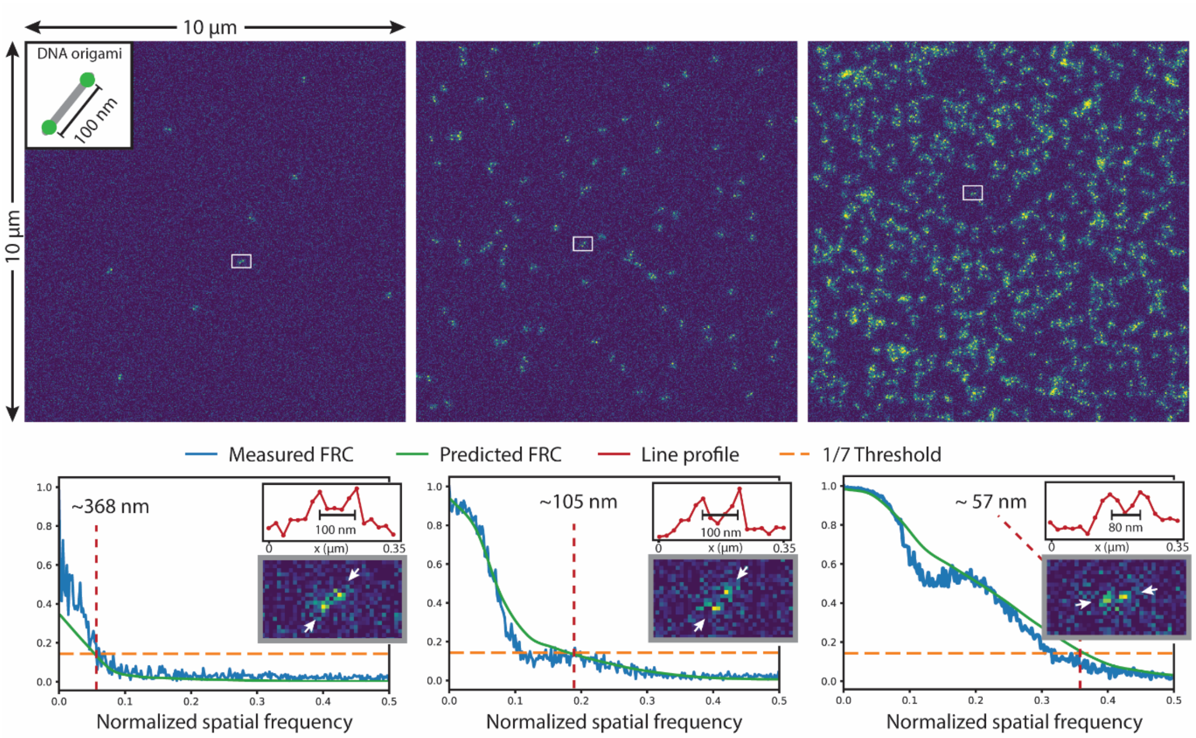

Supplementary Figure 5. Impact of sample density on FRC values. FRC curves reflect how much signal is present in an image of a structure at different frequencies with respect to the noise at that frequency. The value not only depends on the properties of the image formation process, but also inherently on how much of the given frequency is presented in the underlying fluorophore density. If there is no or very little energy in the fluorophore density at a certain frequency, there will surely not be much useful signal in the image at that same frequency. We illustrate this by simulating images of three samples composed by virtual DNA origami structures consisting of two pools of 20 fluorophores each separated by 100 nm. The different samples have a) 10, b) 100 and c) 1000 replicas of these DNA origami structures each in their field of view. The three origami samples roughly have equally shaped spectral profile, although with increasing spectral energy the more origami structures are present. Thus, although the imaging system is exactly the same, the FRC curve will differ vastly and so also the resulting FRC resolution value as defined by the 1/7 threshold. in all three images though, we will claim that the two fluorophore pools can be readily, and equally well, observed as separate points in the three images. The upper row shows the full images. The lower row shows the experimentally measured FRC curves in blue along with the predicted FRC curves for a spectrally flat sample with the same total energy as the imaged sample in green. Yellow dashed line shows the 1/7 threshold. inset shows zooms of highlighted regions along with line profiles on top as indicated by the white arrows.

estimation by reverting the smearing effect of the convolution of the image formation model. These results demonstrate that although great care needs to be taken when interpreting the absolute values of the d_{FRC} metric, the relative improvement or deterioration of d_{FRC} values for images of the same structure does reflect the relative accuracy to which one can measure fine spatial features in images. On the base of these observations, we report the relative d_{FRC} values on the same structure as a measurement of image quality in the following investigations.

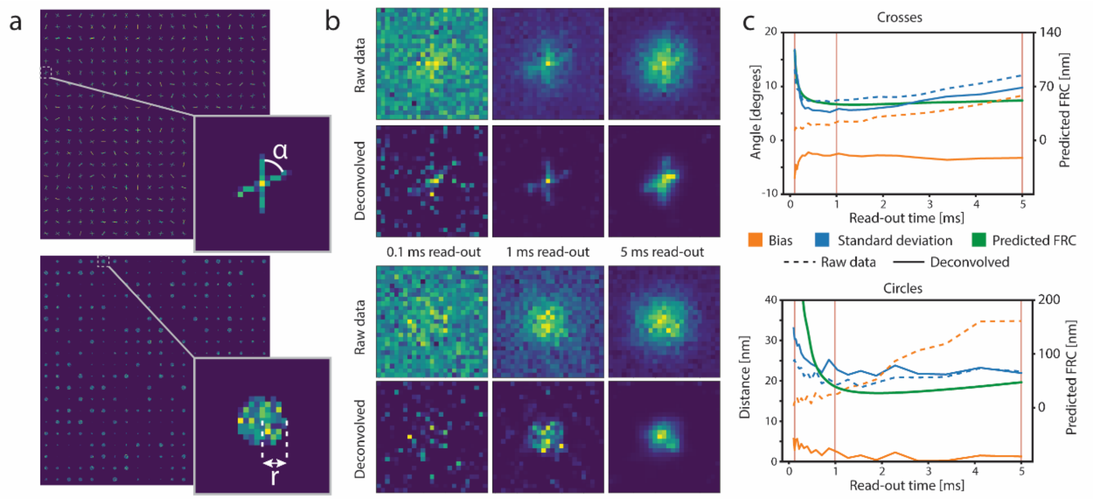

Supplementary figure 6. FRC curves predicts the increase in resolving power. We generate virtual samples consisting of simple structures distributed on a uniform grid. we simulate images of the sample using read-out pulses of different length. Using straightforward and simple quantification algorithms, we quantify properties of the underlying sample from raw and deconvolved images. **(a)** Shows the full images of the virtual samples together with an inset of one of the sub-regions emphasizing the angle α between the lines and the radius r of the circle. **(b)** Shows the same sub-regions as in **(a)** imaged with our simulation tool at different durations of the read-out pulse. Top rows show raw data and bottom rows show deconvolved data. **(c)** Shows standard deviation and bias of the estimations for the raw and deconvolved data at different read-out pulse durations together with the predicted FRC values for the different durations

Quantification algorithm

To justify the use of FRC as a comparative resolution metric, we devised a test to evaluate how well the d_{FRC} value predicts the ability to quantify nanometer scale spatial features in the image. For this we firstly needed to simulate images of virtual samples. Secondly, we needed an algorithm that estimated said spatial features in the images allowing for an objective metric of how well these features could be quantified.

We tried to make the test as simple as possible and created two types of virtual samples, both consisting of a regular grid structure, where each sub-square in the grid contained one replica of either a 200 nm cross with a random crossing angle, or a confined circular region of random radius within a given range.

In the simulated images of these samples, each sub-square was analysed independently by the quantification algorithm which involved the following steps, here with the example of estimating crossing angle:

1. The algorithm is fed with
    a. all relevant parameters used to generate the crosses in the virtual sample, excluding the random crossing angle.
    b. parameters intrinsic for the quantification such as the angular granularity to test.

2. A library of crosses is generated representing all possible combinations of gradually varying line angles.
3. Each generated cross is compared with the imaged cross by calculating the Pearson correlation between the generated cross and the imaged cross.
4. The generated cross having the strongest correlation with the imaged cross is considered the best estimator of the line angles of the imaged cross.

For quantification of the radii of the circular regions, the same approach is used but exchanging the varying crossing angle with a varying radius.

Supplementary Note 3. From experimental parameters to image simulation.

In the prediction software the fluorophore properties and microscope characteristics are inputted by the user to simulate emission kernel, resolution estimation and *in silico* image. To guide the user, we outline here the operation of the software for a specific case, rsEGFP2 imaging in a camera-based parallelized RESOLFT architecture (MoNaLISA[1]). The same analysis pipeline has been followed for all the rsFPs included in the study.

Fluorophore Illumination Response

Given the fluorophore of interest, at each wavelength used for the switching the relative cross section (off-to-on, ON, and on-to-off, OFF cross section) and emission is reported. For a green negative rsFPs like rsEGFP2, 405 nm is used for on switching and 488 nm for off switching and readout. Experimentally, the switching cross sections can be determined by study the power dependence for those wavelengths separately. The cross-section estimation is performed directly on cells to describe the behaviour of the protein in physiological conditions. In this specific case, we used endogenously expressing rsEGFP2 tagged to vimentin, but in general any other construct for which diffusion is not interfering with the kinetics behaviour can be used, for example purified protein embedded in a gel matrix (Supplementary Figure 7).

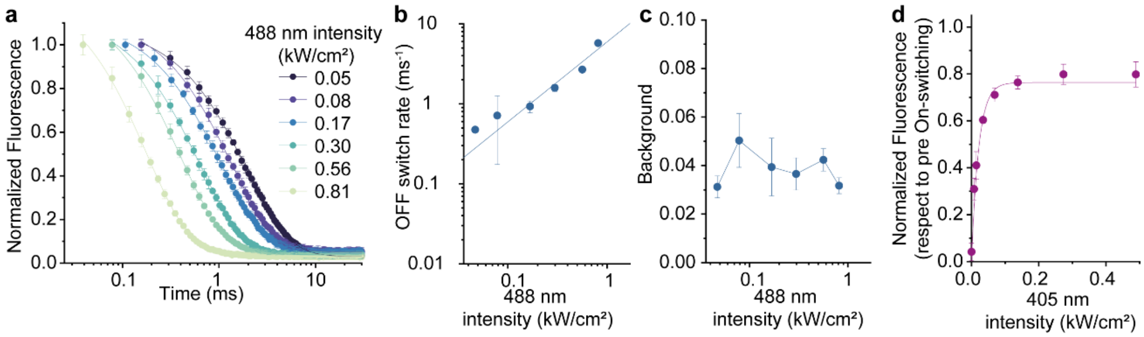

Supplementary Figure 7. Characterization of the switching cross-section for rsEGFP2. (a) OFF-switching decay at increasing intensity of 488 nm light, fitted with an exponential decay with rates following a gamma distribution. Each curve is the mean ± std of at least 3 independent measurements on different cells. (b) Power dependency of the OFF-switching rates (error estimated from the fitted model) and linear fit. (c) Background, i.e. residual fluorescence after prolonged 488 nm light illumination, mean ± std of the last 50 points of the curve in (a). (d) ON-switching curve reporting the increase fluorescence recovered at increased intensity of the 405 nm light. The curve is normalized to the value of fluorescence of the first cycle. Each point is the mean ± std of at least 3 independent measurements on different cells.

As reported in Supp. Fig. 7a for a fixed intensity of the 405 nm on-switching light the decay of fluorescence at different powers of 488 nm light is investigated (from 0.05 to 0.81 kW/cm^2). The off-switching decay fails to be described as a single mono-exponential over the reported power range. Therefore, to account for the complex distribution[2] and still retrieve a single global descriptor for the off-switching that could be inputted in a two-state system we described the

fluorescence signal as an exponential decay with rates following a gamma distribution with parameter α and β, like in [3]:

$$F(t) = \int dk\, e^{-kt}\, P_\Gamma(k;\alpha,\beta) = \frac{\beta^\alpha}{(\beta + t)^\alpha}$$

The off-switching decay can then be described as the time until the signal drops to 1/e:

$$\tau_{1/e} = \beta(e^{1/\alpha} - 1)$$

From this the apparent off switching rate for the 488 nm light can be estimated, $k = 1/\tau = k_{on}^{488} + k_{off}^{488}$. Once combined with the background level, that reflects the equilibrium between the on-to-off and off-to-on switching directions, $F_\infty = k_{on}^{488}/(k_{off}^{488} + k_{on}^{488})$, the specific rates for on and off switching elicited by the 488 nm light can be extracted. The switching cross section can then be determined considering the dependence of the switching rate, k, to the intensity, I:

$$k = \sigma \frac{\lambda}{hc} I$$

Where, λ/hc is the inverse of the photon energy at wavelength λ.

The last parameter to be defined for a given wavelength is the emission constant, ξ ,parameter that correspond to the photon rate divided by the power density. As a simplification this parameter include molecular extinction constant, ε, quantum yield of fluorescence, ϕ, camera conversion factor, δ, and other instrumental loss, η.

For the UV light we make the assumption that this wavelength acts only as off-to-on switch. Therefore, the power dependence of the fluorescence to the 405 nm light intensity can be described as:

$$F(t) = F_0(1 - e^{-k_{on}^{405} t})$$

Where the F_0 represents the percentage of fluorescence that can be recovered saturating the on-switching. The 405 nm light effectively tune the concentration of fluorophore that are actively used during the imaging. More influential on the imaging, increasing intensities of 405 nm will have a strong effect on the fatigue resistance of the protein[4]. According to the absorption spectra the photon budget for this wavelength is set to zero.

Pulse scheme

To reproduce an imaging experiment any temporal sequence of pulses can be designed. Each pulse is defined by the wavelength (among the one specified before), the duration and the intensity at the maxima of an illumination pattern either arbitrary loaded or generated through the program (as Airy spot, doughnut or Gaussian). For a negative switcher like rsEGFP2 the RESOLFT sequence is generally composed of an on-switching pulse that define the local concentration of molecule spatially distributed as a Gaussian profile, followed with a doughnut shaped pulse of blue light to switch back off the molecule in the periphery of the previous beam and finally a Gaussian beam at 488 nm to read out the molecule left in the previously confined sub-diffracted area. The fluorescence used to build the image is always collected from the last pulse. The dimension of the Gaussian and doughnut depends on the specific experimental conditions in use. For example, in case of MoNaLISA recording a periodicity of 312.5 nm is considered for the OFF-switch to match

the experimental setup and the Gaussian FWHM for the 405 and 488 nm beam are 190 and 240 nm, respectively, to match the experimentally measured microlenses focal distribution in the microscope.

Imaging system

The imaging system is described as the optical PSF of the microscope, defined by the numerical aperture and the peak/centre of the emission (for rsEGFP2, 510 nm), as well as the pinhole function in detection, that can be either digital or physical. Different immersion type of objective are considered. The simulation always considers a coordinate-targeted/deterministic switching and reading, therefore different scanning step size can be explored.

Sample properties

The sample is a fundamental aspect of the FRC prediction (Supplementary Note 1). Sample structure with different geometry and density of fluorophore can be loaded. The software can generate three geometries (points, points-pair and lines), that mimic commonly used structure and tool for resolution assessment (i.e. fluorophore beads, DNA-origami nanotemplates, tubulin or filamentous structure in cells). For the image created in Supp. Fig. 8 a binary image drawn to mimic the vimentin sample of reference has been loaded in the software. With a density of fluorophore of 100/µm.

Detector properties

The RESOLFT principle can be integrated in many different architectures that differ in the detection system: point-scanning approach where an APD is used or parallelized approach where an extended camera sensor is needed (i.e. sCMOS). The software account for those different modalities. Both cameras and point detectors can be described by the readout noise (rms) and quantum efficiency. Additionally, the pixel size on the camera needs to be specified for this recording modality. To accurately reproduce the noise level on the camera, the readout noise has to be divided by the conversion factor (electron/count) of the camera. For example, in the simulated image of Supp. Fig. 8, considering the specification of the Orca Fusion (Fast scan modality) used in the experimental system and the magnification set in detection the three parameters are: the readout noise 6.32, resulting from the readout noise (1.35 electrons rms) and the conversion factor (0.22 electrons/count), 65 nm pixel size and 0.8 quantum efficiency.

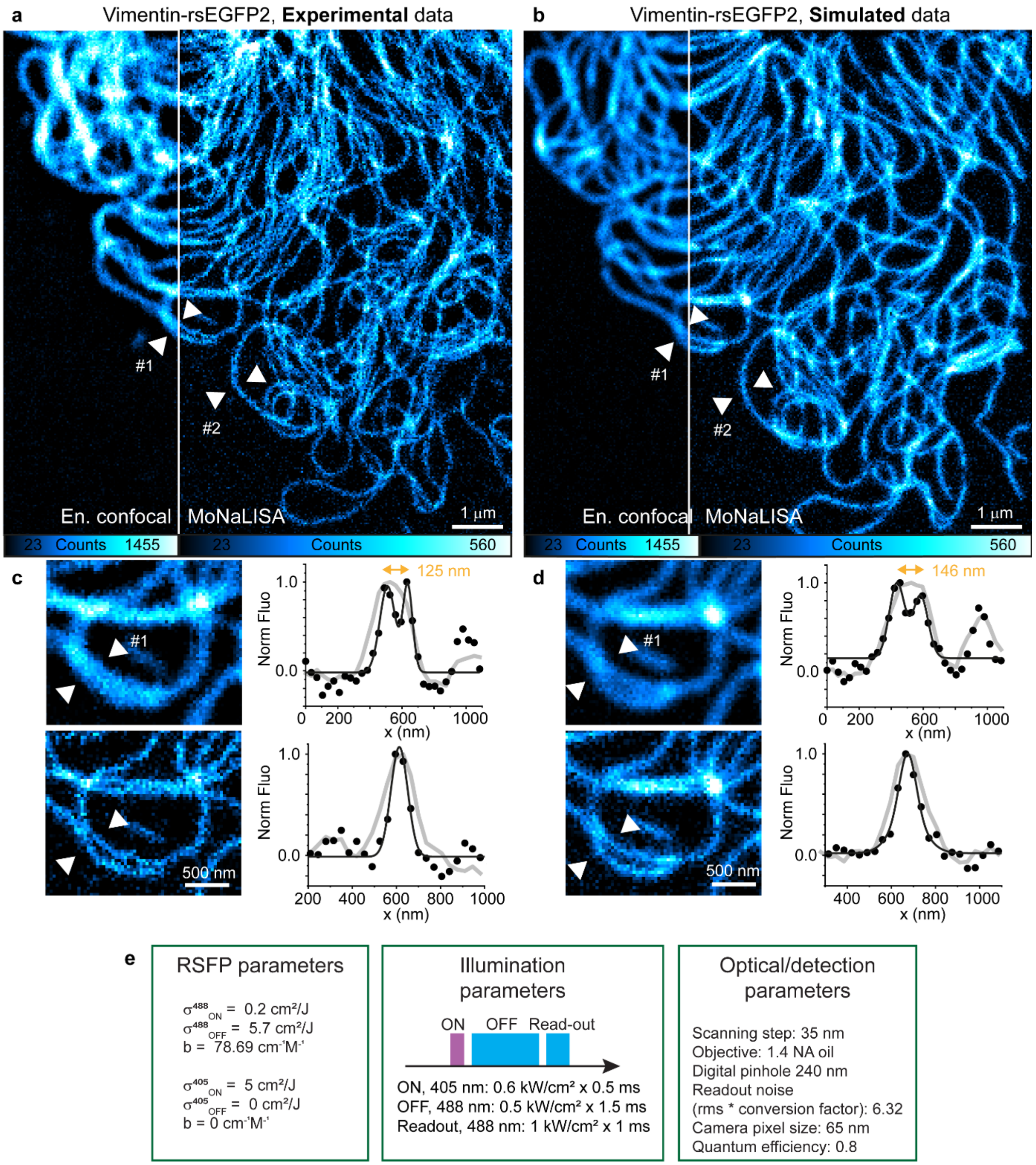

Supplementary Figure 8. Direct comparison of experimental (a) and simulated (b) RESOLFT images. The skeleton of the vimentin-rsEGFP2 of panel a has been used as input image for the simulation in panel b. (c, d) Zoom-in for both images in confocal and RESOLFT mode, with line profile across the region marked in the images (grey lines are experimental confocal, black dots resolft data and solid black lines are fitted Lorentzian over the data). (e) Collection of the software parameters for the generation of panel b.

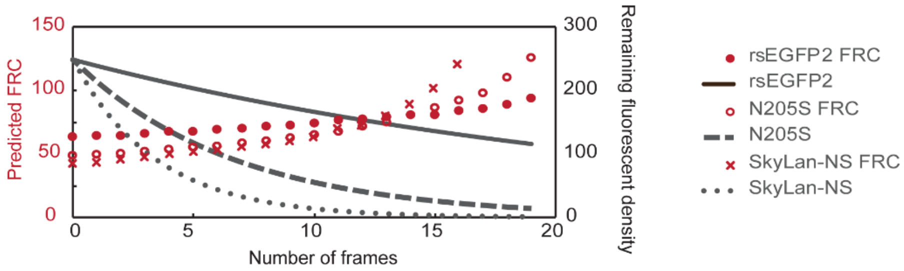

Supplementary Figure 9. Image quality over time by FRC prediction for spectrally flat samples with an exponentially decreasing number of labels on each side.

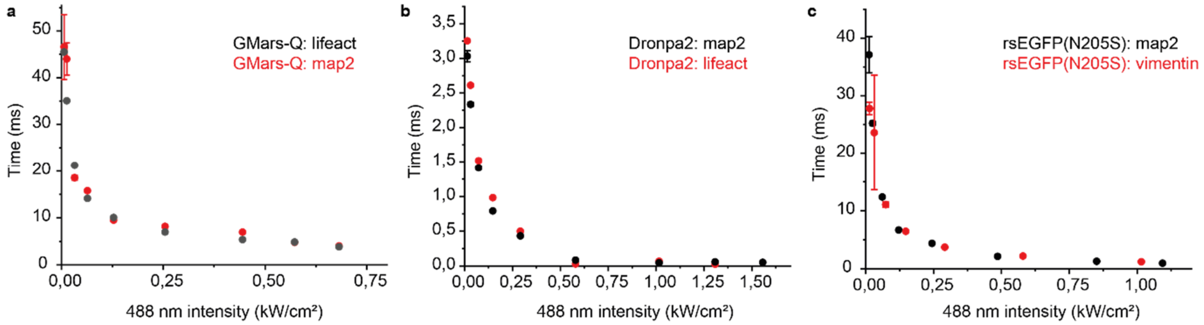

Supplementary Figure 10. Off-switching kinetics for different cytoskeletal construct of GMars-Q, Dronpa2 and rsEGFP(N205S).

Bibliography

(1) Masullo, L. A.; Bodén, A.; Pennacchietti, F.; Coceano, G.; Ratz, M.; Testa, I. Enhanced Photon Collection Enables Four Dimensional Fluorescence Nanoscopy of Living Systems. *Nat Commun* 2018, *9* (1). https://doi.org/10.1038/s41467-018-05799-w.

(2) Nienhaus, K.; Nienhaus, G. U. Chromophore Photophysics and Dynamics in Fluorescent Proteins of the GFP Family. *Journal of Physics Condensed Matter* 2016, *28* (44), 443001. https://doi.org/10.1088/0953-8984/28/44/443001.

(3) Frahm, L.; Keller-Findeisen, J.; Alt, P.; Schnorrenberg, S.; del Álamo Ruiz, M.; Aspelmeier, T.; Munk, A.; Jakobs, S.; Hell, S. W. Molecular Contribution Function in RESOLFT Nanoscopy. *Opt Express* 2019, *27* (15), 21956. https://doi.org/10.1364/oe.27.021956.

(4) Marín-Aguilera, G.; Pennacchietti, F.; Volpato, A.; Papalini, A.; Kulkarni, A.; Bagheri, N.; Minet, G.; Widengren, J.; Testa, I. All-Optical Strategies to Minimize Photo-Bleaching in Reversibly Switchable Fluorescent Proteins. *bioRxiv* 2025, 2025.01.20.633912. https://doi.org/10.1101/2025.01.20.633912.
